# Counting what goes uncounted: strengthening measurement of financial protection in health after the 2025 revision of SDG indicator 3.8.2, with evidence from India

**DOI:** 10.3389/frhs.2026.1955057

**Published:** 2026-10-22

**Authors:** Shyamkumar Sriram

**Affiliations:** Department of Rehabilitation and Health Services, College of Public Affairs and Health Sciences, University of North Texas, Denton, TX, United States

**Keywords:** catastrophic health expenditure, financial protection, health financing, health metrics, India, out-of-pocket payments, SDG indicator 3.8.2, universal health coverage

## Abstract

SDG indicator 3.8.2, the global measure of financial protection in health, is a household-level indicator computed from survey microdata. In March 2025 the UN Statistical Commission replaced its flat thresholds, which under-detected hardship among the poorest households, with a rule that counts out-of-pocket spending above 40% of a discretionary budget net of a societal poverty line, and any spending by households at or below that line. The revised architecture restores, in modified form, the capacity-to-pay approach proposed in 2003: it keeps the 40% threshold and the residual-budget logic, but replaces a food-based subsistence deduction with a poverty line and changes how the poorest households are treated. India's headline statistic, out-of-pocket expenditure (OOPE) as a share of total health expenditure (THE), is a different instrument: a national-accounts ratio of aggregates that says nothing directly about how many households face hardship. It fell to 39.4% in 2021-22, a year the health ministry describes as containing one-off pandemic outlays, and rose to 43.4% in 2022-23. Because government spending sits in its denominator, the ratio can move without any change in household payments; this brief does not establish how much of the movement this explains. India's 2025 national survey reports insurance reaching nearly half the population and a median private-hospitalisation payment of INR 24,000; these are separate population-level statistics, not linked at the individual level. Neither instrument counts households that never seek care, so forgone treatment leaves no trace. Six recommendations follow, for custodian agencies, statistical offices, health ministries and journals; four can be implemented with data already collected.

## 1 Introduction

Financial protection is one of the two goals against which universal health coverage (UHC) is judged, and SDG indicator 3.8.2 is how the world keeps score. Until March 2025 that indicator counted a household as facing hardship when out-of-pocket payments passed a tenth or a quarter of the household budget. A household too poor to spend that much was not classified as facing hardship — which is not the same as being protected, and the difference is the subject of this brief. The world's measure of financial hardship in health was least sensitive precisely where budgets were smallest. The custodian agencies' own review of the indicator put the point empirically: of the roughly one billion people identified in 2019 as spending more than a tenth of their household budget on health, fewer than a quarter were living in poverty on either a relative or an extreme measure, making the identified population pro-rich (1).

In March 2025 the United Nations Statistical Commission, at its 56th session, withdrew the indicator and approved a replacement. The revised measure counts positive out-of-pocket spending exceeding 40% of a household's discretionary budget, meaning consumption or income net of a societal poverty line. Any payment at all by a household at or below that line counts as hardship (2). When the 2025 Global Monitoring Report rebuilt the time series on this basis, the share of the world's population facing financial hardship in 2019 was 12.7 percentage points higher than the superseded measure had implied (3).

The stakes are practical rather than academic. Indicator 3.8.2 anchors national UHC monitoring frameworks and is cited in support of financing reforms that move real money. The revision implies that the population in hardship has been materially larger all along than the official series showed, and that at current rates of progress, 24% of the world's population will still face financial hardship in 2030 (3, 4). What the indicator counts therefore settles which countries appear to be succeeding, which populations appear to need protection, and which reforms appear to have worked.

Three measurement issues run through what follows, at three different levels, and conflating them has caused much of the confusion in this literature (Table 1). The first is household threshold design: where the cut-off sits, and what share of which household budget it is, determines whose spending registers as hardship. This is the problem the 2025 revision addresses (sections 2.1 and 2.2). The second is the aggregate national ratio: out-of-pocket expenditure as a share of total health expenditure is computed from national accounts rather than from households, describes how the national health bill is financed, and contains in its denominator spending set by government (sections 2.3 and 2.4). The third is forgone care: a household that does not seek treatment records no expenditure and is, in the data, indistinguishable from one that faced no hardship; neither of the first two measures can see it (section 2.5). The three are linked. The first two both measure payment, at different levels, so a movement in the aggregate ratio carries no direct information about how many households crossed a hardship threshold. The third measures the complement of both: whatever discourages people from seeking care lowers measured payment at both levels. A falling aggregate ratio is therefore evidence of better protection only when the household indicator does not worsen and forgone care does not rise.

**Table 1:** Three levels at which financial protection is measured.

Level	Instrument	Unit and source	What it can show	What it cannot show	Addressed by the 2025 revision?	Section
1. Household payment against household budget	SDG indicator 3.8.2, superseded and revised	Persons, by household; survey microdata	Whether payments that were made were large relative to what the household had	Hardship among people who did not use care; burdens deferred through borrowing or asset sales	Yes: threshold and denominator redesigned	2.1, 2.2
2. Aggregate payment against national spending	OOPE as a share of THE	Country; national health accounts aggregates	How the national health bill is financed, and the household share of it	How many households face hardship, or which ones; it moves when government spending moves	No: outside the scope of 3.8.2	2.3, 2.4, 2.6
3. Care not obtained	Forgone care conditional on reported illness (proposed companion)	Persons reporting an ailment; survey morbidity module	Whether reported illness went untreated, and the reasons given	Need never perceived as illness, or perceived and not reported	No	2.5, 3

OOPE, out-of-pocket expenditure; THE, total health expenditure.

Revisions on this scale are rare, and they open a window. For a short period the choices buried in a metric become visible and arguable before they settle again. This brief uses that window to put three questions to the agencies, statistical offices and ministries that produce and consume financial protection numbers. What did the revision fix? What did it leave untouched? And what should now be done differently?

### 1.1 Approach and sources

The analysis is documentary and produces no new estimates. Sources were selected purposively rather than systematically, in four groups: the indicator metadata and the accompanying revision documentation from the 2025 SDG Comprehensive Review (1, 2); the WHO and World Bank global monitoring reports on financial protection and UHC (4, 5); India's National Health Accounts (NHA) estimates for 2013-14 through 2022-23 and the National Sample Survey (NSS) 80th round press note (6–14); and the peer-reviewed measurement literature.

Searches covered documents published from January 2003, the year the capacity-to-pay definition was proposed (15), to July 2026. Online sources were last accessed on 30 July 2026, except the National Health Accounts reports for individual years used in Table 4, which were accessed on 3 October 2026. Official documents were retrieved from the websites of the issuing bodies: the UN Statistics Division, WHO, the World Bank, India's Ministry of Health and Family Welfare and its National Health Systems Resource Centre, and the Ministry of Statistics and Programme Implementation. The peer-reviewed literature was identified from the reference lists of those documents and from targeted searches of the measurement literature. Principal search terms were, for the indicator: catastrophic health expenditure, impoverishing health expenditure, financial protection, SDG 3.8.2, capacity to pay, discretionary budget and societal poverty line; for India: out-of-pocket expenditure, National Health Accounts, Ayushman Bharat or PM-JAY, and health insurance, each combined with India; and for forgone care: forgone care, unmet need and self-reported morbidity.

Documents were included if they (i) defined, revised or documented SDG indicator 3.8.2 or its predecessors; (ii) reported the official national aggregates or survey estimates for India on which the argument relies; or (iii) made a methodological contribution to measuring catastrophic or impoverishing expenditure, forgone care or self-reported morbidity, or evaluated the effect of insurance on out-of-pocket payment in India. Studies that only applied an existing definition to report prevalence were not included, because the brief concerns the definitions rather than the estimates.

Conflicting evidence was handled by three rules. Where an official series had been revised, the most recent published round was used, and estimates published as provisional are labelled as such. Where an agency attributed a change to a cause, the attribution is reported as the agency's and is not adopted as a finding. Where peer-reviewed sources disagreed on a question of judgement, as on the merits of capacity-to-pay measures (15, 16), both positions are reported.

All figures quoted are taken from those sources as published. Figure 1 is a schematic constructed by the author from the published indicator definitions; Figure 2 arranges published NHA aggregates; Figure 3 reproduces published NSS estimates. No microdata were analysed and no statistical models were fitted, and the claims made here are correspondingly descriptive and mechanistic rather than causal. India is the worked example: its narrative of falling out-of-pocket expenditure has been prominent, the arithmetic behind it is unusually well documented, and the documents needed to take that arithmetic apart were published by the Indian state itself.

**Figure 1:**
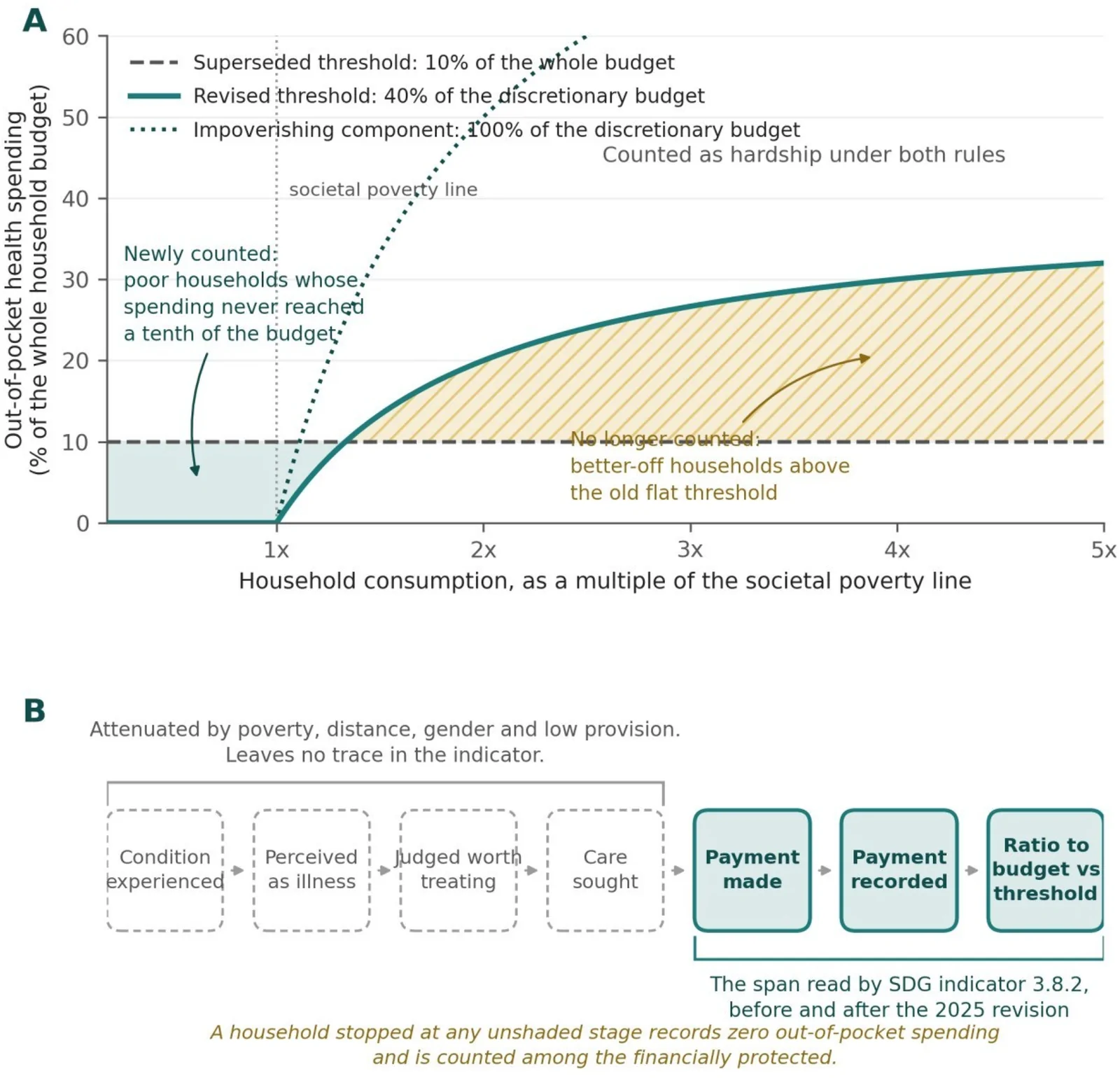
What SDG indicator 3.8.2 counts. **(A)** Households classified as facing financial hardship under the superseded and the revised rule, plotted against household consumption expressed as a multiple of the societal poverty line. The schematic is derived by the author from the published indicator definitions (2) and not from survey data. It assumes a single household size and price level, expresses consumption in multiples of the societal poverty line so that the two rules can be drawn on one axis, and shows the classification boundaries only; it carries no information about how households are distributed along that axis, and the areas shaded should not be read as population shares. The revised boundary is 40% of consumption net of the societal poverty line; the dotted line marks the impoverishing component at 100% of that budget. The architecture restores, in modified form, a capacity-to-pay rule first proposed in 2003; the differences are set out in section 2.2. **(B)** The chain from illness to indicator, showing the span the indicator reads and the earlier stages at which deprivation removes households from the count without registering as hardship, so that such households are not classified as facing financial hardship rather than shown to be protected.

**Figure 2:**
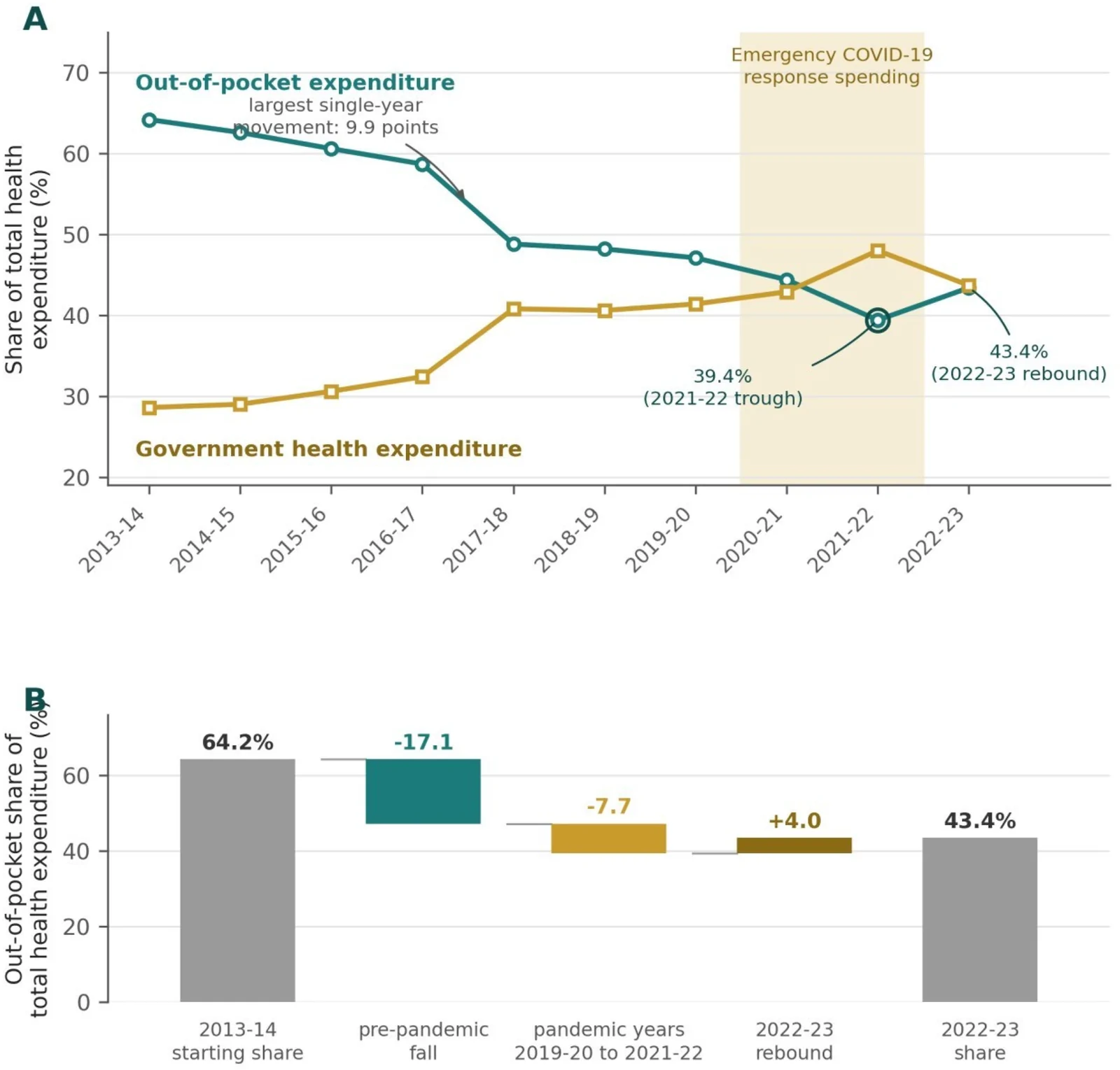
India's out-of-pocket share and the periods in which it moved. **(A)** Government health expenditure and out-of-pocket expenditure as shares of total health expenditure, 2013-14 to 2022-23; the shaded band marks the years of emergency COVID-19 response spending, and the 2020-21 and 2021-22 estimates were published as provisional. **(B)** The movement in the out-of-pocket share partitioned by period, separating the pre-pandemic fall, the pandemic-era fall and the subsequent rebound. Panel B partitions the change in the ratio across time; it does not decompose that change into numerator and denominator contributions, and no causal attribution should be read into it. Absolute values for selected years are given in Table 4. Data from India's National Health Accounts estimates (6–8).

**Figure 3:**
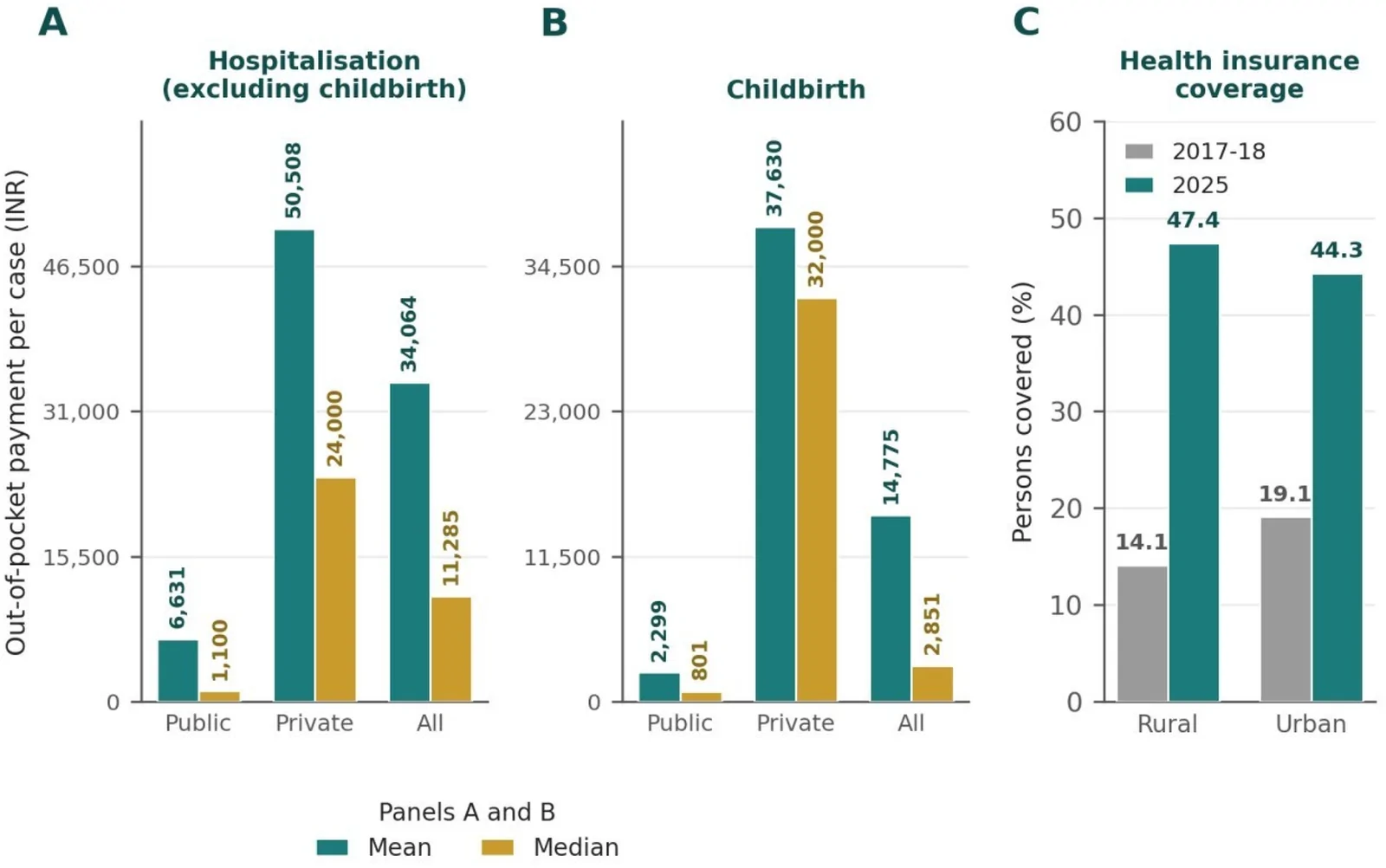
What households pay, and what coverage does not change, India 2025. **(A)** Out-of-pocket payment per hospitalisation case excluding childbirth, and **(B)** per institutional childbirth, by type of provider, showing mean and median. Private hospitals include those empanelled under government financing schemes. **(C)** Percentage of persons covered by any health insurance or financing scheme, 2017-18 and 2025. Panels A and B describe payments among hospitalised persons; panel C describes coverage in the whole population. The three panels are not linked at the individual level: the payment figures are not disaggregated by insurance status, and the panels together show that high coverage and high private-sector payments coexist in the same population, not that the payments shown were made by covered persons. Data from the National Statistics Office, NSS 80th round (9).

## 2 Policy options and implications

### 2.1 What the revision changed

The revision does two things at once. It subtracts a poverty line from the denominator, so the measure asks what a household has left rather than what it has in total, and it replaces a flat threshold with an effective bar that rises across the distribution. Figure 1A shows the two rules on the same plane.

Precisely, let *c* be a household's daily per capita total consumption expenditure (or income), *o* its daily per capita out-of-pocket health payments, and *z* the societal poverty line (SPL). The discretionary budget is *d* *=* *c−z*. Members of the household are counted as facing financial hardship when *o* *>* *0* and *o* *>* *0.4d*. Using 2017 purchasing power parities, *z* is the greater of USD 2.15 a day, the international poverty line, and USD 1.15 a day plus half of median daily per capita household consumption expenditure or income, net of out-of-pocket health spending (2). It therefore has an absolute floor and a relative component that rises with national living standards.

Three features follow from the definition. First, for a household at or below the line, *d* is zero or negative, so the condition *o* *>* *0.4d* is met by any positive payment: such households are counted whenever they pay anything. Second, payments exceeding the whole discretionary budget (*o* *>* *d*), which leave non-health consumption *c−o* below the SPL, form the impoverishing component; payments between 40% and 100% of *d* form the large, non-impoverishing component; the indicator is their sum (2). Third, expressed as a share of total consumption, the effective threshold is *0.4(1−z/c)*: zero at the poverty line, 20% at twice the line, 32% at five times the line, and approaching but never reaching 40% for the best-off households. The indicator is reported as a share of persons, not households.

The result is a measure that identifies poor households whose absolute spending is small, and that no longer identifies better-off households whose spending is proportionally larger but leaves their discretionary budget intact.

This was not a marginal correction. Over six years of accumulating evidence the custodian agencies concluded that a flat tenth of the household budget was too blunt an instrument to register what happens at the bottom of the distribution (4, 17). The superseded rule did not exclude poor households as a matter of definition — a poor household spending more than a tenth of its budget was counted — but it identified them only when their absolute outlays were large enough to clear a bar set as a share of an already small budget, and in practice it identified them much less often than better-off households. Rebuilding the 2019 estimate on the revised basis added 773 million people to the count of those facing financial hardship, against the figure published two years earlier (3). Table 2 sets the two instruments side by side.

**Table 2:** SDG indicator 3.8.2 before and after the 2025 revision.

Dimension	Superseded (2015-2025)	Revised (from 2025)
What is counted	Large out-of-pocket spending, termed catastrophic	Large and impoverishing spending, combined in a single measure
Denominator	Total household consumption or income	Discretionary budget: consumption or income minus a societal poverty line
Threshold	10% and 25% of the budget, applied uniformly	40% of the discretionary budget; any positive spending by households at or below the poverty line
Poorest households	Identified only when spending exceeded a tenth of the whole budget, a bar their budgets made hard to reach; under-detected in consequence	Identified whenever out-of-pocket spending is positive
Richest households	Counted at the same thresholds as everyone else	Counted only above a substantially higher effective bar
Effect on the 2019 global estimate	Reference point	12.7 percentage points higher than the superseded measure implied
Intellectual lineage	Flat budget shares adopted for comparability in 2015	Capacity-to-pay architecture of Xu et al. (2003), modified: a societal poverty line in place of the food-share subsistence deduction (section 2.2)

Sources: United Nations Statistical Division (2); Guo and Grépin (3); WHO and World Bank (4).

Table 3 applies both rules to six hypothetical households in a country where median daily per capita consumption net of out-of-pocket spending is USD 4.00, so that the SPL is the greater of USD 2.15 and USD 1.15 + 2.00, that is, USD 3.15. Household A, below the line, spends USD 0.10 a day on health, 4% of its budget: the superseded rule does not count it; the revised rule does, and classifies the payment as impoverishing. Household E, at almost four times the line, spends 16.7% of its budget: the superseded rule counts it; the revised rule does not, because the payment is less than 40% of its USD 8.85 discretionary budget, an effective bar of 29.5% of its total budget. Households B, C, D and F show the cases in which the two rules agree, and how the revised rule separates impoverishing from non-impoverishing payment.

**Table 3:** Worked example: classification of six hypothetical households under the superseded and revised rules. Values in USD per person per day at 2017 PPP; SPL = USD 3.15.

HH	Consumption c	Out-of-pocket o	o as % of c	Discretionary budget d = c−z	40% of d	Superseded rule (10%)	Revised rule	Revised component
A	2.50	0.10	4.0	−0.65	n/a (*d* ≤ 0)	Not counted	Counted	Impoverishing: already below the SPL
B	4.00	0.30	7.5	0.85	0.34	Not counted	Not counted	–
C	4.00	0.60	15.0	0.85	0.34	Counted	Counted	Large, non-impoverishing (*c−o* = 3.40)
D	4.00	1.10	27.5	0.85	0.34	Counted (also at 25%)	Counted	Impoverishing (*c−o* = 2.90, below the SPL)
E	12.00	2.00	16.7	8.85	3.54	Counted	Not counted	–
F	12.00	4.00	33.3	8.85	3.54	Counted (also at 25%)	Counted	Large, non-impoverishing (*c−o* = 8.00)

Hypothetical values constructed by the author to illustrate the published classification rules (2); not survey data. HH, household; SPL, societal poverty line; PPP, purchasing power parity.

**Table 4:** Out-of-pocket, government and total health expenditure in India, selected NHA rounds (INR lakh crore, current prices).

Year	OOPE	GHE	THE	OOPE, % of THE	GHE, % of THE
2013-14	2.91	1.30	4.53	64.2	28.6
2017-18	2.77	2.31	5.67	48.8	40.8
2020-21	3.28	3.17	7.39	44.4	42.8
2021-22	3.56	4.34	9.04	39.4	48.0
2022-23	3.83	3.85	8.81	43.4	43.7

Values as published in the NHA estimates reports for each year (10–14), rounded to two decimals. One lakh crore = 10^12 rupees. OOPE, out-of-pocket expenditure; GHE, government health expenditure; THE, total health expenditure.

### 2.2 A return as much as an advance

What the announcement did not dwell on is that the new architecture is not new. Xu and colleagues proposed, in 2003, that catastrophic health expenditure be defined as out-of-pocket payments exceeding 40% of income remaining once subsistence needs had been met, and built the first multicountry estimates on that basis (15). Wagstaff and van Doorslaer set out the rival family of measures the same year (18).

When the SDGs were operationalised in 2015, that capacity-to-pay approach was set aside in favour of flat budget shares, which were simpler to compute, easier to compare across countries and less dependent on a contestable subsistence line (19, 20). In the interval Wagstaff argued that capacity to pay does not in fact establish whether payments are large enough to undermine a household's ability to buy non-medical necessities (16). The 2025 revision returns to that approach in modified form, with a societal poverty line standing in for the subsistence deduction.

The return is architectural rather than literal, and the differences are not cosmetic. The two definitions share three features: the 40% threshold; the logic of judging payment against a residual budget left after basic needs, rather than against the whole budget; and total household consumption, including out-of-pocket spending, as the starting point. They differ in at least four. First, the deduction. Xu and colleagues subtracted a household-specific subsistence amount derived from food spending: the average food expenditure per equivalent adult among households whose food share lay between the 45th and 55th percentiles of the national distribution, scaled by an equivalence factor of household size raised to the power 0.56. The 2025 indicator subtracts a per capita poverty line defined in purchasing power parity terms, chosen because a single definition applied across countries preserves comparability (2, 15). Second, households below the line. In the 2003 method, a household whose actual food spending fell below the subsistence amount had its capacity to pay computed as total spending minus actual food spending; capacity to pay stayed positive, and the household was counted only if its payments passed the 40% test. Under the 2025 rule any positive payment by such a household counts. Third, impoverishment. The 2003 multicountry analysis reported catastrophic expenditure; impoverishment was measured separately in the literature that followed (18). The 2025 indicator folds impoverishing payment into a single measure and reports it as a component. Fourth, the unit: the 2003 estimates are shares of households, the 2025 indicator a share of the population.

What carries over, then, is the threshold and the residual-budget logic. How the residual is drawn, and how sensitive the resulting estimates are to that choice, does not; the two definitions will not in general classify the same household the same way.

So the sequence runs: proposed in 2003, set aside in 2015 for comparability, criticised on its merits in 2019, restored in modified form on equity grounds in 2025. That sequence is not easily read as convergence on a truer number. It is more readily read as a record of which value was carrying most weight at each juncture — comparability in 2015, equity in 2025, both defensible on the evidence available at the time. That reading is an interpretation of the outcomes, offered here as a way of understanding them; we have not examined the deliberative records, and the participants may describe their reasoning differently. Treating headline indicators as settlements between competing values, rather than as facts, is not scepticism; it is a well-established description of how such instruments are made (21, 22).

### 2.3 When the denominator is policy-sensitive

Turning to India requires one distinction first, because the object of measurement changes. Indicator 3.8.2 is a household-level measure built from survey microdata. The statistic India reports, out-of-pocket expenditure (OOPE) as a share of total health expenditure (THE), is a national accounts ratio built from aggregates. They are not the same instrument, and evidence about one is not evidence about the other. What they share is a habit: each expresses household payment as a fraction of something that is not the household's experience of paying. For 3.8.2 the denominator is at least the family's own budget. For the accounts ratio it is everyone's health spending, the state's included, which belongs to the family not at all — and which contains components set by the same authorities that publish the ratio. The further up that ladder a measure sits, the easier it becomes to move the number without moving anything for any household.

Successive NHA rounds reported OOPE falling from 64.2% of THE in 2013-14 to 39.4% in 2021-22, presented as evidence that financial protection was arriving (6, 7). Then the estimates for 2022-23, released in May 2026, put the share back at 43.4% (8). Figure 2 shows the shape, and Table 4 gives the numerator and denominator behind the shares for selected rounds.

Read as levels rather than shares, the published aggregates show something the ratio hides. In current prices, household out-of-pocket spending rose in every year shown from 2017-18 onward, including the trough: from INR 3.28 to 3.56 lakh crore between 2021-22 and 2021-22 (8.5%), and to INR 3.83 lakh crore in 2022-23 (7.4%). The share fell in 2021-22 because the denominator grew faster: THE rose by 22.3% and government health expenditure by 37.2%. It rose in 2022-23 because government health expenditure fell by 11.2% and THE by 2.6% while household spending continued to grow.

The ministry's own release attributes the earlier trough to the denominator. Government health spending had been lifted to 1.84% of gross domestic product in 2021-22 to fund emergency COVID-19 response packages and the national vaccination campaign, and the release presents that one-time spending as the reason the share fell to 39.4% (8).

That attribution is the ministry's. Table 4 is consistent with it, but this brief does not establish its magnitude, for four reasons. The figures are nominal and national; they are neither deflated nor expressed per person, so they do not show whether real household payments per person rose or fell. They are aggregates, so they do not show which households paid. The NHA out-of-pocket series is itself an estimate rather than an annual observation of household payments: for 2022-23 it draws on the NSS 75th round health survey of 2017-18 and the 2022-23 Household Consumption Expenditure Survey (10), so a change in the share combines movements in an administratively recorded denominator with a modelled numerator. And no counterfactual is estimated here: what household or government spending would have been without the emergency outlays is not known. What the published series supports is narrower and still worth stating. The fall to the trough and the subsequent rebound coincide with the arrival and withdrawal of extraordinary public outlays; the ministry identifies those outlays as the cause of the trough; and the aggregates move in the direction a denominator effect would produce. The two pandemic years account for 7.7 of the 24.8 percentage points of the total decline, and 4.0 points returned the following year — arithmetic properties of the published series, not attributions of cause.

The series warrants care in any case: its largest single-year movement, 9.9 percentage points between 2017-18 and 2017-18, precedes the pandemic entirely, and the 2020-21 and 2021-22 estimates were published as provisional. Most of the decade's decline predates the pandemic. Whatever the trough turns out to represent, it is the part of the series least able to bear weight.

The obvious word here is gaming, and it is the wrong one. Nobody had to spend in order to move the number. The reactivity described in the sociology of quantification, in which the measured rearrange themselves around the measure (23), requires actors who know the metric, care about it and adjust to it. None of that is needed here. A ratio whose denominator contains components set by policy can move for reasons unconnected to the experience it summarises, with everyone acting in good faith and nobody looking at the indicator at all. This failure mode leaves no strategic behaviour to document and yields the same misleading headline. It also requires no intent, and therefore no unusual conditions — though how often it occurs relative to deliberate gaming is an empirical question this brief cannot answer.

### 2.4 Coverage is not payment

What households actually pay is settled elsewhere. The NSS 80th round, fielded through 2025 and released in April 2026, put the average out-of-pocket payment per hospitalisation excluding childbirth at INR 6,631 in a public facility and INR 50,508 in a private one (9). That private category expressly includes hospitals empanelled under government financing schemes. For childbirth, near-universal in institutions at 96.2% of births, the gap is wider still: INR 2,299 in public facilities against INR 37,630 in private ones (Figures 3A,B). Where a household is treated bears heavily on what it pays, and no national share can see the difference.

The distributions matter as much as the averages. Across all hospitals the mean payment was INR 34,064 and the median INR 11,285; in public hospitals the median was INR 1,100, and for outpatient care in public facilities it was zero, meaning half of those episodes cost nothing (9). A mean carried by a long tail and a median close to the floor describe different populations, and a threshold rule applied to household microdata will find a different picture again. An indicator built on one of these and read alongside the others will mislead.

Coverage, meanwhile, is not the same thing as protection. Health insurance enrolment in India rose from 14.1% of rural and 19.1% of urban people in 2017-18 to 47.4% and 44.3% in 2025, most of it through government-sponsored schemes (9) (Figure 3C). That more-than-threefold rise in rural enrolment coexists with a median payment of INR 24,000 across all private hospitalisations excluding childbirth, by insured and uninsured patients, rural and urban, in a category that includes empanelled hospitals. These two facts are population-level statistics from the same survey round, published separately, and they are not linked at the individual level. Their coexistence shows that, at current levels of enrolment, large private-sector payments remain common among hospitalised persons. It does not show that insured people made those payments, and the INR 24,000 median should not be read as the median payment of an insured household. What can be said is that enrolment figures on their own carry no information about payment, and are routinely read as though they did.

For the individual-level question the appropriate evidence is evaluation, and it points the same way: four years into the Ayushman Bharat Pradhan Mantri Jan Arogya Yojana, enrolment was not associated with lower out-of-pocket or catastrophic spending, and contracted private hospitals went on charging patients (24). The direct evidence would be simpler still. Payments disaggregated by insurance or scheme status and by provider type would settle the question within a single survey round; the NSS collects what is needed, and the published tabulations do not include the cross-tabulation. Recommendation R5 in section 3 asks for this reporting as a matter of routine.

A further distortion runs underneath all of this. Payments met by borrowing or by selling assets enter the accounts as ordinary expenditure, displacing the burden into a future the survey window never reaches (25).

### 2.5 The limb that is still not measured

One limitation survives the revision untouched. Financial protection is officially achieved when there are no financial barriers to access and when the payments required do not cause hardship (5). Indicator 3.8.2 measures the second condition only, because it is built from what households spent. A household that does not seek care records nothing, sits at zero in the numerator and joins those not classified as facing hardship. Forgone care, the most complete failure of financial protection there is, enters the statistic on the same side as its opposite.

In India that silence has a documented shape, and the newest data make it starker. The NSS 80th round found 13.1% of people reporting an ailment in a fifteen-day window in 2025, against 7.5% in the 2017-18 round (9).

An underlying near-doubling of India's disease burden over seven years is not plausible, but the comparison should be read with care in both directions. The two rounds differ in fielding period, in questionnaire and in context; the 2017-18 round preceded the pandemic, and post-pandemic reporting of symptoms and contact with the health system may differ for reasons unrelated to either burden or access. The change most likely reflects some combination of greater recognition and reporting of illness, wider contact with the health system, and differences between the instruments themselves. It is a change in reported illness, and the direction of the underlying change in need is not established by it.

Within 2025 the gradient also runs the wrong way for anyone reading morbidity as a measure of need. Urban residents reported more illness than rural residents, 14.9% against 12.2%, while rural residents used public facilities more often when they did seek care.

Judgements about one's own health are positional, formed by what a person has learned to expect and to notice, so populations with the least provision may report the least morbidity (26, 27). Self-reported morbidity is therefore a fallible measure of need in both directions: it can understate need where expectations are low, and overstate it where medical contact is dense. An episode must be experienced, perceived as illness, judged worth treating, treated, paid for and recorded before it reaches the indicator, and deprivation thins that chain at its earliest links (Figure 1B).

What would a companion measure require? Less than might be assumed. Most national household surveys already ask whether a person reporting an ailment sought treatment, and why not if they did not. The missing element is not an instrument but an obligation to publish those responses alongside the expenditure indicator, disaggregated on the same dimensions, so that a rising financial protection score can be checked against whether more people are getting care or fewer. Where the two move in opposite directions, the protection signal is likely to be an artefact of withdrawal from the system.

The reasons people give for not seeking care fall into groups with different meanings, and the companion measure is useful only if it keeps them apart. Financial barriers are the failure of financial protection proper. They include the cost of treatment, and also indirect costs such as transport and lost earnings, which are financial in effect but do not appear in out-of-pocket health spending. Other access barriers, such as no facility within reach, long waits or lack of confidence in the care available, are failures of service coverage, the other half of SDG target 3.8, rather than of financial protection. A judgement that the ailment did not need treatment may be correct, or may reflect the low expectations described above, or a decision to avoid cost that the respondent does not describe as financial. The headline companion statistic should therefore be the share of persons reporting an ailment who did not obtain care for financial reasons, with the other reasons reported beside it rather than pooled into it. A fall in financial reasons accompanied by a rise in others is a different story from a fall in all forgone care, and a single all-cause rate cannot tell them apart. Even the financial category will undercount: respondents may cite distance or the seriousness of the ailment when cost was decisive, so the financially attributed rate is best read as a lower bound.

Such a measure would be a partial companion, and its limits should be stated with it. It is conditional on an ailment being reported: it would capture people who recognised an illness and did not obtain care, together with the reasons they gave. It would not capture need that was never perceived as illness, or perceived and never reported — the earliest links in the chain in Figure 1B, and precisely the links that deprivation thins most. Nor would it be free of the reporting problems set out above, since its own denominator is self-reported morbidity. It is proposed as a companion to 3.8.2 rather than a solution to the problem 3.8.2 cannot address, and it would move the boundary of what is observable outward by one stage rather than removing it.

### 2.6 Beyond India

The mechanism described in section 2.3 is not specific to India as a matter of arithmetic, but this section states a property of the measure, not an empirical finding. Any country reporting out-of-pocket spending as a share of total health expenditure is exposed to it, because part of the denominator is set by policy. Where public spending rises sharply for reasons unconnected to household circumstances — a large one-off external grant, an emergency response to an epidemic or a disaster — the ratio falls, other things equal, and rises again when the outlay ends. Whether that dominates any particular observed movement depends on what happened to household payments over the same period, which the ratio does not reveal. We have not examined any other country's series, and this brief offers no evidence that the mechanism has affected any country other than India; its empirical claims are confined to India.

One setting where the possibility could be tested is suggested by the contraction of development assistance for health after 2025. In countries where external financing forms a large share of public health spending, a deterioration in the ratio over the next reporting cycles could reflect a denominator returning to trend as much as worsening protection. That is a hypothesis to be checked against real per-capita household payments, not a prediction.

This analysis has three limits. First, it is documentary and estimates no quantity itself. Table 4 reports the published aggregates in nominal terms, but the change in the share is not decomposed into numerator and denominator contributions in real per-capita terms, and the counterfactual out-of-pocket share India would have recorded without emergency spending is not calculated; the mechanism is described and its magnitude is not established. Second, the NHA estimates for 2020-21 and 2021-22 were published as provisional, and the out-of-pocket series is itself estimated from survey rounds that are not annual, so the trough may move again. Third, the survey statistics used in section 2.4 are population-level and cannot speak to the association between insurance and payment within individuals; the evaluation literature is cited for that purpose and carries its own limitations. None of the three bears on the recommendations below, which turn on how numbers are reported rather than on their exact values.

## 3 Actionable recommendations

Table 5 sets out six recommendations, with the actors responsible, the data each requires, the output it would produce for monitoring, and whether it can be implemented with data already collected.

**Table 5:** Actionable recommendations: responsibilities, data requirements and monitoring outputs.

No.	Recommendation	Lead (and contributing) actors	Data required	Monitoring output	Existing data sufficient?
R1	Publish dual-vintage estimates through the transition	WHO and World Bank as custodian agencies (national statistical offices supply microdata)	Household survey microdata already used to compute 3.8.2	Superseded and revised series for overlapping years, published together, with the break stated	Yes – core action
R2	Develop and monitor a companion indicator of forgone care, conditional on reported illness, separating financial from other reasons	WHO and World Bank to specify; national statistical offices to compute and publish	Survey morbidity module: ailment reported, treatment obtained, reason if not; consumption for quintiles	Financially attributed forgone-care rate, with other reasons alongside, by wealth quintile, sex and residence, recall period stated	Partly: questions exist in many surveys, including the NSS; a harmonised definition is needed
R3	Report the impoverishing and non-impoverishing components of the revised indicator separately	National statistical offices; custodian agencies in the global database	Same microdata as 3.8.2	Two component shares reported beside the headline total	Yes – core action
R4	Publish real per-capita OOP and government health spending alongside the OOP share, and flag years containing one-off outlays	Health ministries and NHA technical secretariats (in India, the National Health Systems Resource Centre); statistical offices for population and price series	NHA nominal OOPE and GHE; mid-year population; consumer price index or health deflator	Constant-price per-capita OOPE and GHE series, with deflator, base year and population series named, one-off years flagged	Yes – core action
R5	Publish insurance enrolment and out-of-pocket payment for the same population and period, cross-tabulated by insurance status and provider type	National statistical offices (in India, the National Statistics Office); health ministries	Unit-level survey records with insurance or scheme status, provider type and payment per episode	Mean and median payment per episode by insurance status and provider type	Yes – core action: collected, not yet tabulated
R6	Require every empirical claim about catastrophic health expenditure to state threshold, denominator, indicator vintage and survey instrument	Journals, editors and peer reviewers	None	Reporting requirement in author guidelines and review checklists	Yes

OOP, out-of-pocket; OOPE, out-of-pocket expenditure; GHE, government health expenditure; NHA, National Health Accounts.

Four of the six recommendations, R1, R3, R4 and R5, are tabulations of data already collected and could be implemented in the next reporting cycle; they are the core actions. R6 requires only editorial policy. R2 requires the most work: the survey questions exist, but a common definition of the numerator, the reason categories and the recall period has to be agreed before estimates are comparable across countries or survey rounds. The reasoning behind each recommendation follows.

The transition itself needs managing (R1, R6). Series are being rebuilt on the revised definition while a decade of published research, national reporting and programme evaluation rests on the superseded one. For several years two vocabularies will circulate under one name, and estimates that differ by more than twelve percentage points will be compared as though they measured the same thing (3). Custodian agencies should publish dual-vintage series through the transition, and every empirical claim about catastrophic health expenditure should state its threshold, denominator, indicator vintage and survey instrument. Journals are well placed to require those four items, and the burden on an author is a single sentence.

Financial protection has two components in the official definition and only one in the official measure (R2). A companion indicator should be specified as follows, so that it can be built from instruments already in the field. Headline numerator: persons who reported an ailment in the recall window, did not obtain treatment for it, and gave a financial reason — the cost of treatment, or indirect costs such as transport or lost earnings. Secondary numerators: the same persons by each non-financial reason (no facility within reach, waiting or quality concerns, ailment judged not to need treatment, other). Denominator: persons who reported an ailment in the same recall window. Recall period: that of the host survey's morbidity module — fifteen days for outpatient episodes and 365 days for hospitalisation in the Indian instrument — stated explicitly with every estimate, since ratios computed over different recall lengths are not comparable. Disaggregation: wealth or consumption quintile, sex, and rural or urban residence at minimum, matching the disaggregation published for 3.8.2. Reporting: the headline and secondary ratios together, since “no need felt” and “could not afford” carry opposite policy implications and averaging over them destroys the signal. Stated limitations: the headline ratio is a lower bound, because cost-driven decisions may be reported under other reasons, and it is conditional on an ailment being reported, so it does not observe unperceived or unreported need. Until such an indicator is monitored alongside 3.8.2, no country can show that a rising score reflects protection rather than withdrawal — and even with it, the demonstration is partial.

A ratio reported on its own can move for reasons that have nothing to do with households, as India's did (R4). The figure that resolves the ambiguity is real out-of-pocket spending per person: nominal OOPE from the national accounts divided by the mid-year population, deflated to a stated base year by a stated index — the consumer price index, or a health-specific deflator where a credible one exists — and reported in constant national currency, with the index, the base year and the population series named alongside the estimate. Government health expenditure per capita should be reported on the same basis, and years containing one-off outlays should be flagged in the series. Published alongside the share, these two levels let a reader see immediately whether a falling ratio reflects households paying less or the state paying more. India's NHA reports already publish nominal per-capita figures; what is missing is the constant-price series and the flag on one-off years.

Enrolment figures and payment figures currently travel separately, and enrolment travels further (R5). India now reports insurance reaching nearly half its population alongside a median payment of INR 24,000 for a private hospitalisation. Published in separate documents by separate agencies, the first reads as though it had settled the second. Reporting them together, for the same population and period, would stop that. Reporting them cross-tabulated — payment per episode by insurance or scheme status and by provider type — would go further and answer the question the two separate figures only raise. This is a tabulation of data already collected, not a new instrument.

The cheapest recommendation is R3. Reporting the impoverishing and non-impoverishing parts of the revised indicator adds one line to a table, and it separates a country in which hardship is deepening among those already poor from one in which it is spreading to households above the poverty line. Those are different problems calling for different instruments, and a single headline figure hides which one a country has.

## 4 Conclusions

This brief makes three kinds of statement, and they should be read differently.

**What it shows, from published documents.** The 2025 revision of SDG indicator 3.8.2 changed the threshold and the denominator of the global measure of financial protection, so that payments are judged against what a household has left after a societal poverty line, and any payment by a household at or below that line counts. Rebuilding the 2019 estimate on that basis added 773 million people to the count of those facing financial hardship (3). The revised rule restores, in modified form, the capacity-to-pay architecture proposed in 2003: it shares the 40% threshold and the residual-budget logic, and differs in how the residual is drawn and in how households below the line are treated. In India, the national-accounts ratio of out-of-pocket to total health expenditure, a different instrument from 3.8.2, reached its low point in 2021-22, a year in which government health expenditure rose by 37% and household out-of-pocket spending continued to rise in nominal terms; the ministry attributes the trough to one-off pandemic outlays. Insurance enrolment and private-hospitalisation payments are published as separate population-level statistics and are not linked at the individual level. Reported illness rose between the 2017-18 and 2025 survey rounds, a change the instruments do not allow to be read as a change in need.

**What it proposes.** Six reporting changes, four of which use data already collected: dual-vintage series through the transition; the impoverishing and non-impoverishing components reported separately; real per-capita out-of-pocket spending published beside the share; and payments cross-tabulated by insurance status and provider type. A companion indicator of forgone care, conditional on reported illness and separating financial from other reasons, would need agreed definitions. Journals can require every claim about catastrophic expenditure to state threshold, denominator, vintage and instrument.

**What it hypothesises or leaves open.** That reading the 2003 to 2025 sequence as a settlement renegotiated describes it better than convergence on a truer measure is an interpretation, not a finding from the deliberative record. That the denominator mechanism accounts for a substantial part of India's 2021-22 trough is consistent with the published aggregates but is not quantified here. That the same mechanism will move the ratio in countries where external health financing contracts is a possibility to be tested, not an observation.

The three reporting streams are complements, not substitutes. Aggregate ratios, reported with their numerator and denominator in real per-capita terms, show how the national health bill is financed. Household-level indicators show whether the payments that were made caused hardship. Monitoring of forgone care shows who did not pay because they did not get care. None is sufficient alone, and a headline from any one of them can move in the wrong direction while the others reveal why. None of this is hidden; each element sits in the documents that generate the numbers. Four questions put to any claim of financial protection would go a long way: who is counted, at what threshold, against which denominator, and who has to be silent for progress to appear. None of the four needs access to microdata. All four can be put to the press release.
